# A few antibiotics can represent the total hospital antibiotic consumption

**DOI:** 10.1186/s12879-018-3132-7

**Published:** 2018-05-31

**Authors:** Bongyoung Kim, Hyeonjun Hwang, Jieun Kim, Myoung-jae Lee, Hyunjoo Pai

**Affiliations:** 1 Department of Internal medicine, College of Medicine, Hanyang University, 222-1 Wangsimni-ro, Seongdong-gu, Seoul, 04763 South Korea; 2 School of Economic Sciences, Washington State University, Pullman, USA; 3 Department of Economics, College of Political Science & Economics, Korea University, 145 Anam-ro, Sungbuk-gu, Seoul, 02841 South Korea

**Keywords:** Antibiotic consumption, antimicrobial stewardship, statistical model, fluoroquinolone, aminoglycoside

## Abstract

**Background:**

Appropriate antibiotic use has become an important issue. However, collecting data on the use of all antibiotics in a hospital is difficult without an advanced computerized system and dedicated staff. This paper examines if 1–3 antibiotics can satisfactorily represent the total antibiotic consumption at the hospital level.

**Methods:**

We collected antibiotic data from six large university hospitals in Korea for some years between 2004 and 2012. Since the total antibiotics consist of a few chosen representative antibiotics and the rest, we used those chosen antibiotics along with additional variables constructed only with *t* (time) such as *t*, *t*^*2*^, and *t*^*3*^ to capture the time trend and whether *t* belongs to each month or not to capture the monthly variations. The ordinary least squares method was used to explain the total antibiotic amount with these variables, and then the estimated model was employed to predict the use for 2013. To determine which antibiotics were the most representative in tracking general trends in antibiotic use over time, we tried various combinations of antibiotics to find the combination that best minimized the 2013 prediction error.

**Results:**

We found that fluoroquinolones and aminoglycosides were the most representative, followed by beta-lactam/beta-lactamase inhibitors and 4th-generation and 3rd-generation cephalosporins. The mean prediction error over 12 months in 2013 with these few antibiotics was only 1–3% of the monthly antibiotic consumption amount.

**Conclusions:**

The total antibiotic consumption amount at the hospital level can be represented sufficiently by a few antibiotics, such as fluoroquinolones and aminoglycosides, which means that hospitals can save resources by tracing only the usage of those few antibiotics instead of the entire inventory. Since the choice of fluoroquinolones and aminoglycosides is based solely on our Korean data, other hospitals may follow the same modelling methodology to find their own representative antibiotics.

**Electronic supplementary material:**

The online version of this article (10.1186/s12879-018-3132-7) contains supplementary material, which is available to authorized users.

## Background

Antimicrobial resistance is a worldwide problem, which poses a serious threat to global public health [1]. In 2011, to prevent further worsening of the problem, the World Health Organization (WHO) urged nations to be alert for antimicrobial resistance and called for urgent action to decrease antimicrobial consumption [2]. In accordance with the initiative, the Korean government launched a national action plan on antimicrobial resistance in 2016 [3].

As is well documented, the overuse/misuse of antibiotics has been recognized as a key factor for the emergence of antimicrobial-resistant organisms [4]. Inappropriate antibiotic use also causes extra medical expenses: unnecessary or duplicative antibiotic use in US hospitals led to an estimated $163 million in excess costs [5]. Hence, many experts have suggested establishing antimicrobial stewardship programmes in hospitals as well as in communities [1]. The first step to combat antibiotic abuse is finding out the severity of the problem. This calls for a proper measurement of antibiotic consumption [6], which helps to understand the epidemiology of antimicrobial resistance and provides hospitals with useful data to implement policies and guidelines about proper antibiotic usage [6].

To this end, we collected data on antibiotic prescriptions from six large Korean university hospitals with good computerized systems. There were considerable difficulties in collecting data on antibiotics because there were too many different types of antibiotics but no experienced/dedicated staff to collect data on antibiotics. There was no problem obtaining data on the prescription department, administration route and volume of drug, but a big problem in the data collection was that antibiotics were recorded by the brand name, not by the ingredient names nor by the antibiotic class name. This required extra effort to convert the data into a suitable and consistent form.

The goal of this paper is to explore whether it is possible to look at only a couple of representative antibiotics to determine the total antibiotic consumption at the hospital level. If yes, this means a considerable savings in terms of time and effort to keep track of all antibiotic use. To this goal, we built a statistical model, in which 1–3 representative antibiotics are chosen to predict the total antibiotic consumption at the hospital level, with an acceptably small magnitude of prediction error.

## Methods

### Study design

We build a simple linear statistical model, where the total antibiotic consumption at the hospital level is explained by 1–3 representative antibiotics along with time and month dummy variables–the time and month dummy variables are “free”, as they depend on time index *t* only. Because the total amount consists of the representative antibiotics and the remaining (non-representative) ones, this modelling strategy amounts to explaining the remaining antibiotics using their correlations with the representative antibiotics as well as the time and month dummy variables. We estimate the model using the observations over 2004–2012 of six large university hospitals in Korea, one of which is the Hanyang University Seoul Hospital (HUS). Then, the model prediction capability is evaluated for the ensuing year (2013), using the data from HUS.

### Data source

We collected data on the total antibiotic prescriptions for inpatients and their total patient days in 2004, 2008 and 2012 from six university hospitals (4 tertiary and 2 secondary) in Korea: Hanyang University Seoul Hospital (758 beds), Chungbuk University Hospital (620 beds), Chonnam University Hospital (970 beds), Gyeongsang University Hospital (889 beds), Hanyang University Guri Hospital (578 beds), and Korea University Ansan Hospital (543 beds). In addition, we collected data from HUS for each year between 2004 and 2013 on the total antibiotic prescription records and the total patient days. All data were extracted from the electronic billing system by the data processing department in each hospital.

### Definitions

We define antibiotics as medications with class J01 in Anatomical Therapeutic Chemical (ATC), which does not include antifungal agents nor anti-tuberculosis agents. Systemic agents with oral or parenteral administration routes are included, but topical agents are excluded. We convert each class of antibiotic amount to a defined daily dose (DDD) by using the ATC of the WHO and then standardize for 1000 patient days [7].

We classify antibiotic agents into 19 classes: 1st-generation cephalosporins (1st CEP), 2nd-generation cephalosporins (2nd CEP), 3rd-generation cephalosporins (3rd CEP), 4th-generation cephalosporins (4th CEP), aminoglycosides (AG), beta-lactam/beta-lactamase inhibitors (BL-BLI), carbapenems, fluoroquinolones (FQ), glycopeptides, lincosamide, macrolides, metronidazole, monobactam, oxazolidinone, penicillins, polymyxin, tetracyclines, tigecycline and trimethoprim/sulfamethoxazole. Other antibiotics such as amphenicol, fosfomycin, and streptogramin are excluded because they are rarely used.

Let *n*_*ht*_ denote *the patient days for hospital h* = 1 … 6 and *month t*; *t* ranges over 1 … 12 (year 2004), 49 … 60 (year 2008) and 97 … 108 (year 2012) for the hospitals other than HUS, and *t* ranges over *t* = 1 … 108 for HUS. Let *DDD*_*aht*_ denote the *DDD for antibiotics a, hospital h, and time t*. With ‘≡’ standing for “defined as”, let ‘all-hospital patient days at time t’ and ‘all-hospital DDD for antibiotics a at time t’ be

$$ {n}_t\equiv \kern0.5em {n}_{1t}\kern0.5em +\kern0.5em \dots \kern0.5em +\kern0.5em {n}_{6t}\kern0.5em and\kern0.5em {DDD}_{at}\equiv {DDD}_{a1t}+\kern0.5em \dots \kern0.5em +\kern0.5em {DDD}_{a6t} $$

Then, the all-hospital DDD per 1000 patient days for antibiotics a at month t is

$$ {X}_{at}\kern0.5em \equiv \frac{DDD_{at}}{n_t}\kern0.5em \times \kern0.5em 1000. $$

Let *m* (‘*m*’ for main) be the number of the main (i.e., representative) antibiotics; *m* = 1, 2 or 3 in this paper. Listing the main antibiotics first, the total antibiotic amount at time *t* can be written as

1 $$ {Y}_t\kern0.5em \equiv \kern0.5em \mathrm{the}\ \mathrm{main}\ \mathrm{antibiotic}\ \mathrm{amount}+\kern0.5em \mathrm{the}\kern0.5em \mathrm{others}\kern0.5em =\kern0.5em \sum \limits_{a=1}^m{X}_{at\kern0.5em }+\kern0.5em \sum \limits_{a=m+1}^{19}{X}_{at} $$

### Statistical Methodology

To achieve our goal of representing the total *Y*_*t*_ with the main antibiotics, it is necessary to account for the remaining part ${\sum}_{a=m+1}^{19}{X}_{at}$ in (1) in a simple way. We achieved this by replacing the sum ${\sum}_{a=m+1}^{19}{X}_{at}$ with “free variables”. If the free variables can represent ${\sum}_{a=m+1}^{19}{X}_{at}$ well enough, then we do not have to collect data on those remaining antibiotics.

We used three types of free variables to account for ${\sum}_{a=m+1}^{19}{X}_{at}$: (i) time index *t* to capture the trend, (ii) month dummies to capture the monthly variations, (iii) and some calendar time dummies to capture “structural breaks” (i.e., big events), if there are any. Since all three types are determined by *t*, no data collection is needed for them. We illustrate these three types next. Let 1[*A*] = 1 if *A* holds, and 0 otherwise.

Suppose we have *t* = 1 … 17 monthly observations over January 2004 to May 2005. First, use a polynomial function such as ${\sum}_{q=0}^p{\alpha}_q\kern0.5em {t}^q$(e.g., ${\sum}_{q=0}^2{\alpha}_q\kern0.5em {t}^q={\alpha}_0\kern0.5em +\kern0.5em {\alpha}_1t\kern0.5em +\kern0.5em {\alpha}_2{t}^2$) to account for the trend, where α s are the parameters to be estimated using (1, *t* …*t*^*p*^). Second, capture the monthly variations with the month dummies; e.g., the February dummy 1[*t* ∈ *February*] is to capture the February effect relative to the baseline January, where ‘∈’ means “belonging to”, and the March dummy 1[*t* ∈ *March*] is to capture the March effect relative to January. Third, there might be a big policy change, say, a crackdown on antibiotic abuse at *t* = 6 and onwards, in which case 1[6 ≤ *t*] can be used to account for the crackdown that is a structural break.

Since the main antibiotics and *t*-based variables are in the model, whereas the other antibiotics are not, in essence, the omitted non-representative antibiotics are explained by their correlations with the main antibiotics and the *t*-based variables. After the model parameters are estimated using the time-series data up to *t* = 108 (December 2012), we then construct the predicted *Y*_*t*_ for *t* = 109~ 120 (2013) for HUS using the estimated model; let ${\widehat{Y}}_t$ denote the predicted value.

After model estimation using *t* = 1~ 108, predicting ${\widehat{Y}}_t$ for *t* = 1~ 108 is “in-sample prediction”, and predicting ${\widehat{Y}}_t$ for *t* = 109~ 120 is “out-sample prediction”. The out-sample prediction is to pick the representative antibiotics, and the in-sample prediction is just to see how the chosen representative antibiotics perform in fitting the in-sample observations. Since we put more emphasis on predicting the future than on explaining the past, the out-sample prediction is our primary criterion to determine the representative antibiotics, whereas the in-sample prediction is secondary.

To explain how to select 1–3 main antibiotics, suppose *m* = 2. For each main antibiotic candidate, we obtain ${\widehat{Y}}_{109},\kern0.5em ..\kern0.5em {\widehat{Y}}_{120}$ for 2013 and its “mean prediction error”:

2 $$ \frac{1}{12}\kern0em \sum \limits_{\tau =109}^{120}\left|{Y}_{\tau}\kern0.5em -{\widehat{Y}}_{\tau}\right| $$

In other words, (2) is the average of the monthly absolute deviations for 2013 between the actual and predicted antibiotic uses in DDD/1000 patient days. The particular combination of two antibiotics minimizing (2) is the best choice.

To explain why we consider different values for *m*, the reason is that there is a trade-off in setting *m* large v. small. If *m* is large, say 10, we can trace the overall antibiotic consumption better, but then the representativeness will be worse; if *m* is small, say 1, then the opposite happens. Between these extremes, 1–3 seem to be reasonable values, and for each chosen value of *m*, we try different combinations of antibiotics.

The model for the ordinary least squares (OLS) estimator, where the main antibiotic amount and the above “free” *t*-based variables collectively explain *Y*_*t*_, is shown in Additional file 1: Tables S3 and S4; Additional file 1: Table S3 uses all six hospitals’ data, whereas Additional file 1: Table S4 uses only the HUS data for the model estimation. In each table, the OLS estimates and their standard errors (SE) are provided. Dividing an estimate by its SE gives the ‘t-value’ or ‘z-score’. It being above 2 in absolute value indicates statistical significance at the 5% error level, i.e., we set statistical significance at *P* < 0.05. *R*^2^ shows the proportion of the *Y*_*t*_ variation explained by all “regressors” (i.e., explanatory variables) jointly.

## Results

### Most prescribed antibiotics in the pooled data

Pooling all time-series data of the six hospitals into one big data set, Table 1 provides descriptive statistics in all six hospitals, including HUS, as well as HUS alone; the unit for all numbers is DDD/1000 patient days. The average total antibiotic consumption of the six hospitals plus/minus the standard deviation (SD) is 864 ± 55.5, and that of HUS is 915 ± 100. Overall, 3rd CEP was used most in all six hospitals (24.8% from 213.82/862.94), followed by FQ (12.1%, 104.11/862.94), 2nd CEP (11.4%, 98.17/862.94), 1st CEP (10.6%, 91.84/862.94) and BL-BLI (10.5%, 90.27/862.94). Similarly, 3rd CEP (18.8%, 173.26/920.69) was used most in HUS, followed by 1st CEP (15.5%, 143.10/920.69), FQ (13.4%, 123.15/920.69), 2nd CEP (12.7%, 116.94/920.69), AG (9.8%, 90.57/920.69) and BL-BLI (8.0%, 74.11/920.69). In contrast, monobactam, oxazolidinone and tigecycline were rarely used; there was even no use at all of these antibiotics for some months.

**Table 1:** Antibiotic consumption in all hospitals and in only Hanyang University Seoul hospital (unit: DDD/1,000 patient days)

	Six hospitals (2004, 2008, 2012 : 36 months)		Hanyang University Seoul hospital (2004-2012 : 108 months)	
	Mean (SD)	Range^a^	Mean (SD)	Range^a^
1^st^ CEP	91.7 (8.8)	78.5-114.0	142.0 (23.4)	93.7-210.0
2^nd^ CEP	98.4 (21.9)	60.8-138.0	115.0 (31.7)	77.9-204.0
3^rd^ CEP	214.0 (15.4)	188.0-249.0	172.0 (19.6)	136.0-231.0
4^th^ CEP	11.4 (5.8)	3.73-21.0	14.6 (9.7)	0-38.0
AG	71.2 (47.4)	19.5-146.0	86.6 (56.2)	18.3-184.0
BL-BLI	90.2 (7.4)	74.8-101.0	74.9 (15.8)	44.1-123.0
Carbapenems	14.7 (5.4)	6.4-24.6	9.9 (5.7)	0-22.1
FQ	104.0 (7.0)	90.3-121.0	123.0 (14.2)	88.6-172.0
Glycopeptides	23.3 (2.1)	18.3-27.2	18.7 (4.6)	9.3-32.4
Lincosamide	16.6 (3.8)	10.7-23.8	15.6 (4.9)	4.6-31.2
Macrolides	41.8 (8.1)	29.4-59.3	44.8 (14.6)	21.3-88.2
Metronidazole	41.0 (7.8)	30.6-61.9	43.6 (7.7)	21.3-61.2
Monobactam	0.6 (0.5)	0-2.0	0.8 (1.0)	0-4.5
Oxazolidinone	0.7 (0.6)	0-2.1	1.1 (1.1)	0-4.8
Penicillins	20.4 (6.4)	10.1-37.7	17.6 (7.5)	2.0-41.2
Polymyxin	2.8 (2.1)	0-6.3	2.3 (2.6)	0-11.6
Tetracyclines	9.7 (6.4)	3.5-30.9	4.7 (4.6)	0-21.7
Tigecycline	0.3 (0.5)	0-1.7	0.7 (1.2)	0-4.8
Trimethoprim/sulfamethoxazole	11.2 (1.9)	8.1-17.1	26.2 (10.1)	5.8-51.5
Total	864.0 (55.5)	766.0-975.0	915.0 (100.0)	770.0-1121.0

^a^Monthly consumption averaged over the years

Abbreviations: *1*^*st*^*CEP* 1st-generation cephalosporins, *2*^*nd*^*CEP* 2nd-generation cephalosporins, *3*^*rd*^*CEP* 3rd-generation cephalosporins, *4*^*th*^*CEP* 4th-generation cephalosporoins, *AG* aminoglycosides, *BL-BLI* beta-lactam/beta-lactamase inhibitors, *FQ* fluoroquinolones

### Out-sample prediction, representative antibiotics, and in-sample fitness

Table 2 shows the main antibiotics minimizing the mean prediction error (2). For example, the mean prediction error is 26.2 DDD/1000 patient days using only AG for *m* = 1, and it is 17.2 using AG and 4th CEP for *m* = 2, where the predictors were obtained with all six hospitals’ data. In contrast, the mean prediction error is 20.7 DDD/1000 patient days using only FQ for *m* = 1, and it is 18.3 using FQ and AG for *m* = 2, where the predictors were obtained with only the HUS data.

In Table 2, when all six hospitals’ data are used in the left half, AG does best (with the mean prediction error 26.2 when used alone), followed by FQ, 4th CEP, and BL-BLI. When only the HUS data are used, FQ or BL-BLI does best, followed by AG and 3rd CEP. Combining these findings, we may state that FQ is the most representative, followed by AG, BL-BLI, 4th CEP and 3rd CEP. Since the total number of observations is 180 (=36 months times 5 hospitals) plus 108 (12 months times 9 years from HUS) and HUS takes only 37.5% of the total observations 288 = 180 + 108, the result based on the entire data set that AG is the best changes when only the HUS observations are used. The details on the OLS used for prediction are provided in the Additional file 1.

**Table 2:** Representative antibiotics and mean prediction error (unit: DDD/1,000 patient days)

	Six hospitals		Hanyang University Seoul hospital (HUS)	
# main antibiotics	Representative antibiotics	Prediction error	Representative antibiotics	Prediction error
*m* = 1	AG	26.2	FQ	20.7
	FQ	26.9	BL-BLI	22.0
*m* = 2	AG + 4^th^ CEP	17.2	AG + FQ	18.3
	AG + penicillins	18.1	BL-BLI + FQ	18.7
*m* = 3	AG + BL-BLI + 4^th^ CEP	12.3	BL-BLI + FQ + 3^rd^ CEP	15.0
	AG + 4^th^ CEP + monobactam	14.0	AG + BL-BLI + 3^rd^ CEP	15.4

Abbreviations: *3*^*rd*^*CEP* 3rd-generation cephalosporins, *4*^*th*^*CEP* 4th-generation cephalosporins, *AG* aminoglycosides, *BL-BLI* beta-lactam/beta-lactamase inhibitors, *FQ* fluoroquinolones

Define the “mean prediction error multiplied by 100 and divided by the monthly antibiotics consumption amount in Table 1” as the “relative mean prediction error”. Using the six hospitals’ data, the relative mean prediction error for *m* = 1, 2, and 3 is, respectively,

$$ \frac{26.2}{864}\kern0.5em \times \kern0.5em 100\kern0.5em =3.0\%,\kern0.5em \frac{17.2}{864}\kern0.5em \times 100\kern0.5em =2.0\%,\kern0.5em \frac{12.3}{864}\kern0.5em \times 100\kern0.5em =1.4\% $$

Using the HUS data, the relative mean prediction error for *m* = 1, 2, and 3 is

$$ \frac{20.7}{915}\kern0.5em \times 100\kern0.5em =2.3\%,\kern0.5em \frac{18.3}{915}\kern0.5em \times 100\kern0.5em =2.0\%,\kern0.5em \frac{15.0}{915}\kern0.5em \times \kern0.5em 100\kern0.5em =1.6\% $$

Judging from the *m* = 2 and 3 cases here and the rows for *m* = 2 and 3 in Table 2, although the predicted *Y* is for HUS, using all the hospital data is preferable to using only the HUS data to minimize the prediction error.

**Fig. 1:**
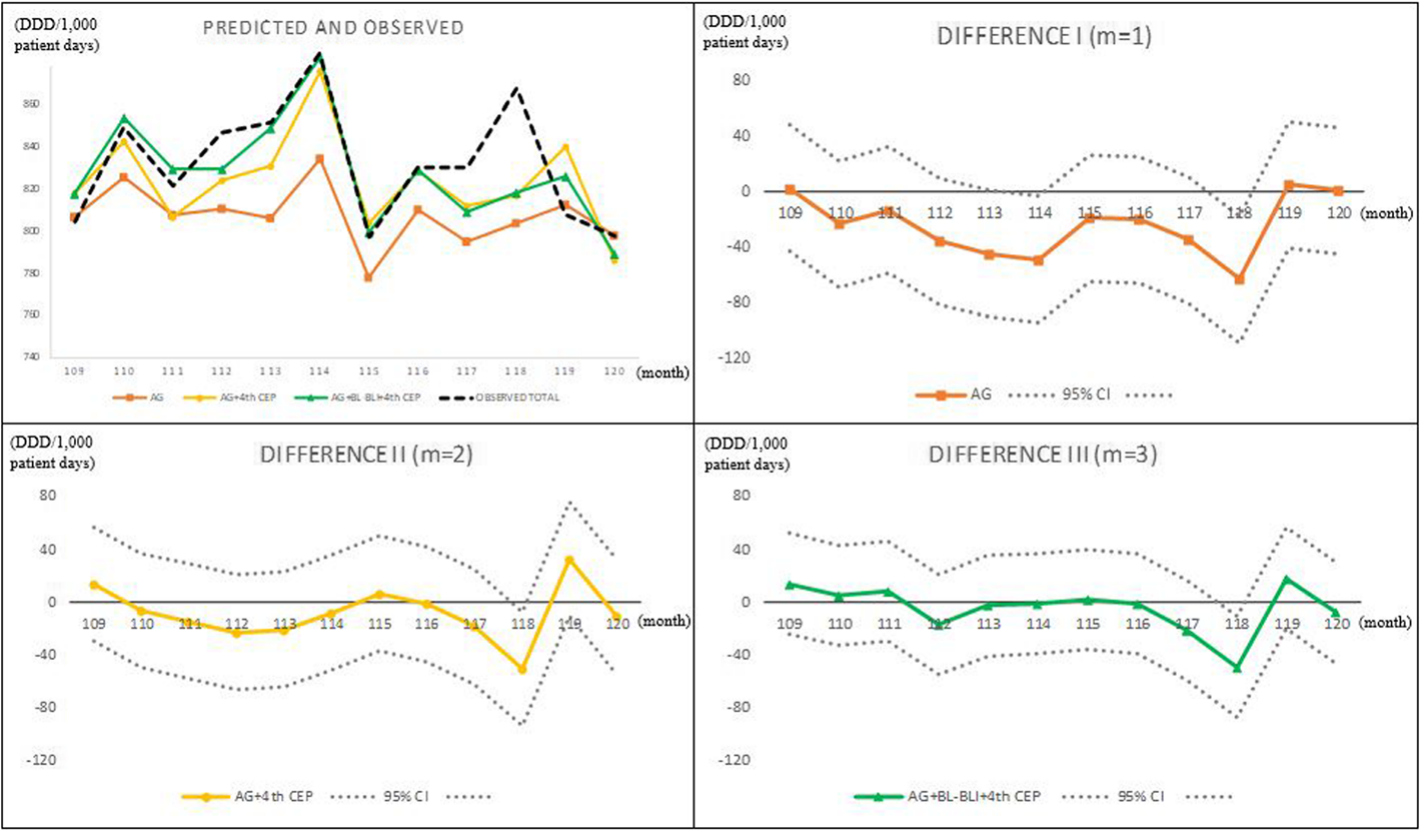
Out-sample prediction for Hanyang University Seoul Hospital, 2013, using six hospital data sets. Abbreviations: *4th CEP* 4th-generation cephalosporins, *AG* aminoglycosides, *BL-BLI* beta-lactam/beta-lactamase inhibitors, *CI* confidence interval

**Fig. 2:**
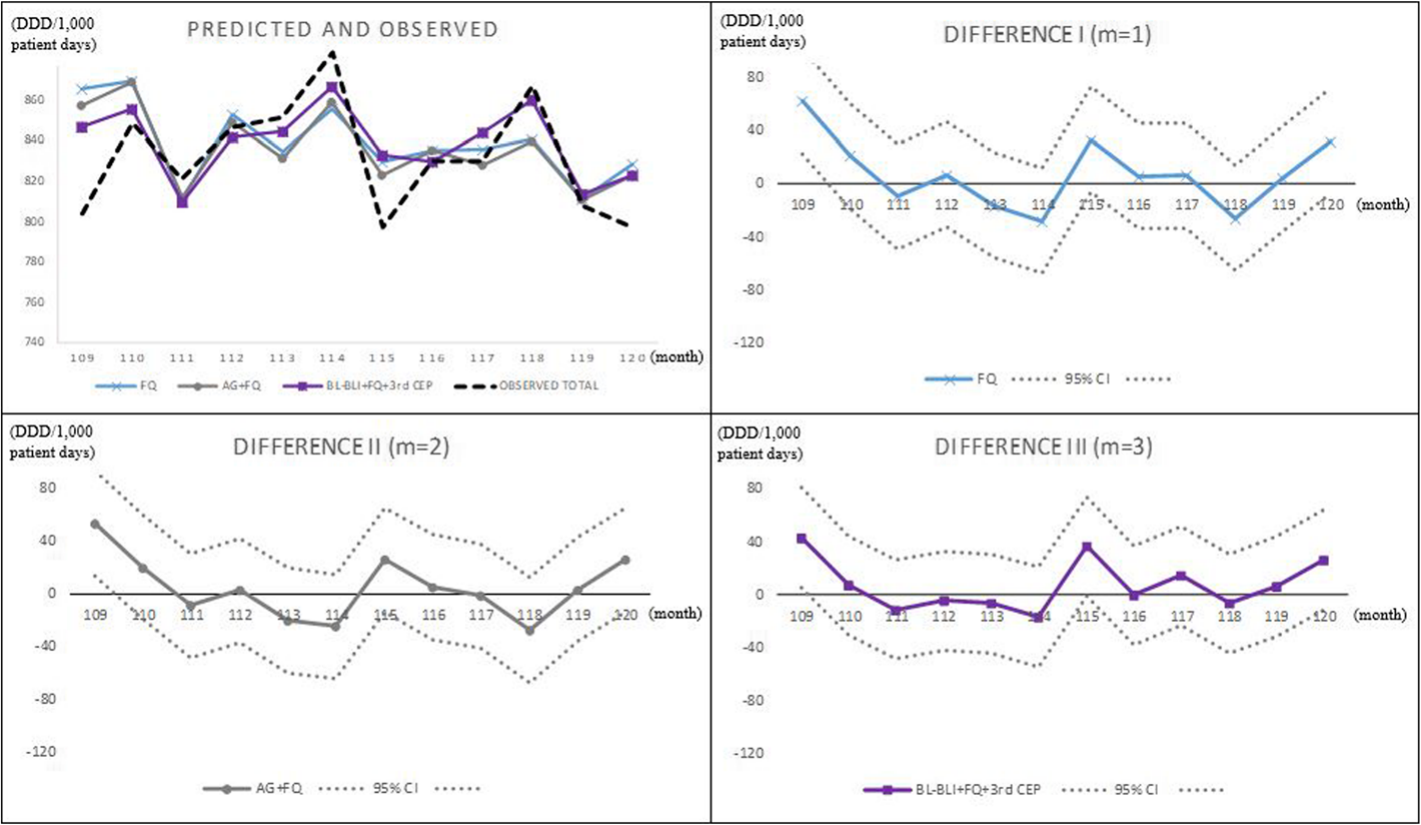
Out-sample prediction for Hanyang University Seoul Hospital, 2013, using Hanyang University Seoul Hospital data only. Abbreviations: *3rd CEP* 3rd-generation cephalosporins, *AG* aminoglycosides, *BL-BLI* beta-lactam/beta-lactamase inhibitors, *FQ* fluoroquinolones, *CI* confidence interval

Figure 1 shows the out-sample prediction time-series plot and the difference between the observed and predicted values using all hospital data, and Fig. 2 shows the same using only the HUS data. The figures show that the predicted lines match the actual line (dotted) well, and the 95% confidence interval for the prediction error includes zero in almost all cases.

**Fig. 3:**
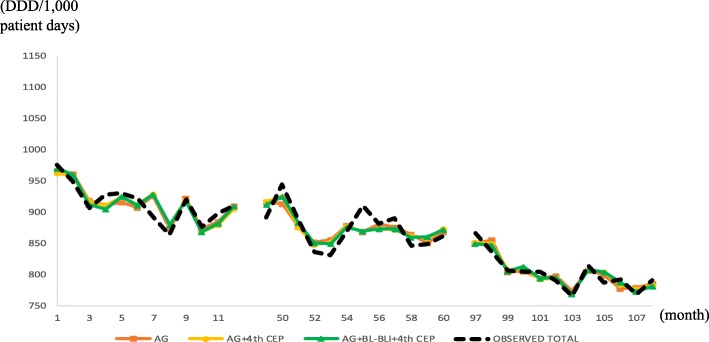
In-sample prediction (2004, 2008, 2012) for six hospitals, using six hospitals’ data. Abbreviations: *4th CEP* 4th-generation cephalosporins, *AG* aminoglycosides, *BL-BLI* beta-lactam/beta-lactamase inhibitors

**Fig. 4:**
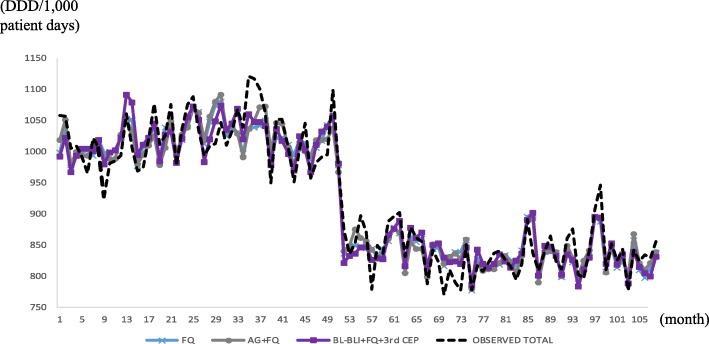
In-sample prediction for Hanyang University Seoul Hospital, using Hanyang University Seoul Hospital data only. Abbreviations: *3rd CEP* 3rd-generation cephalosporins, *AG* aminoglycosides, *BL-BLI* beta-lactam/beta-lactamase inhibitors, *FQ* fluoroquinolones

Figure 3 presents the in-sample actual and fitted (*m* = 1,2,3) values for the six hospitals, and the fitness looks good; Fig. 4 does the same for HUS. Notice a large drop at *t* = 52 in Fig. 4, which prompted using 1[52 ≤ *t*] in the OLS for the HUS data.

### The most prescribed antibiotics are not necessarily the most representative

**Fig. 5:**
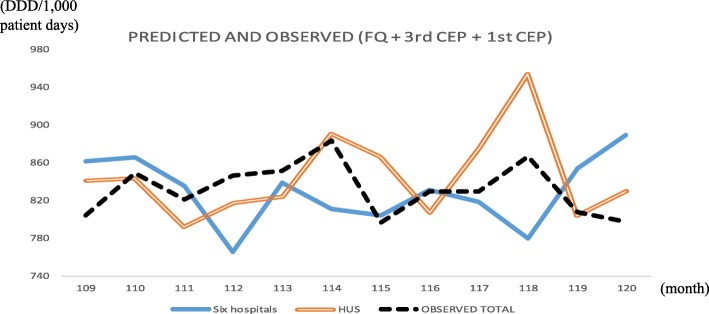
Out-sample prediction for Hanyang University Seoul Hospital, 2013, with three most commonly used antibiotics (FQ, 3rd CEP, and 1st CEP). Abbreviations: *FQ* fluoroquinolones, *3rd CEP* 3rd-generation cephalosporins, *1st CEP* 1st-generation cephalosporins

**Fig. 6:**
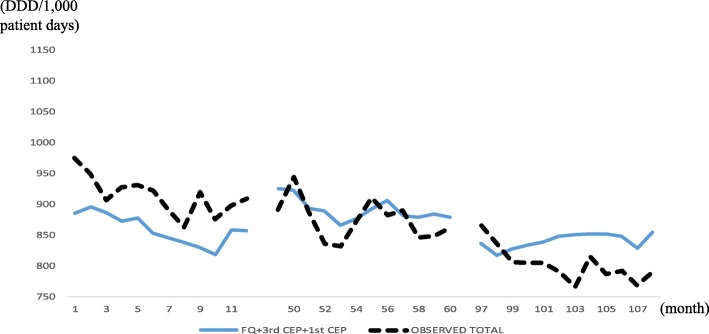
In-sample prediction (2004, 2008, 2012) for six hospitals with the most commonly used antibiotics (FQ, 3rd CEP, and 1st CEP). Abbreviations: *FQ*, fluoroquinolones *3rd CEP* 3rd-generation cephalosporins, *1st CEP* 1st-generation cephalosporins

**Fig. 7:**
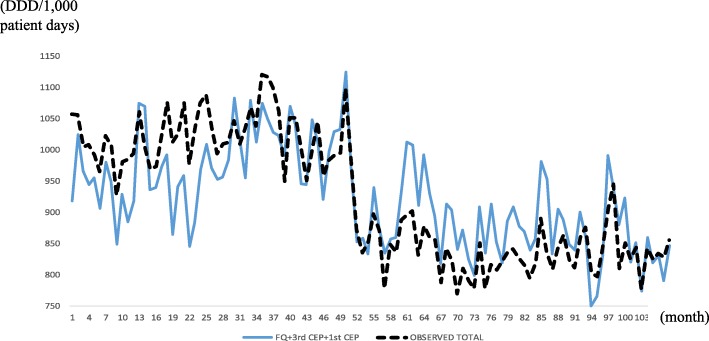
In-sample prediction for Hanyang University Seoul Hospital with the most commonly used antibiotics (FQ, 3rd CEP, and 1st CEP). Abbreviations: *FQ* fluoroquinolones, *3rd CEP* 3rd-generation cephalosporins, *1st CEP* 1st-generation cephalosporins

Taking the most prescribed antibiotics (FQ, 1st CEP and 3rd CEP) as the three representative antibiotics, we redrew Figs. 1, 2, 3 and 4 to present the result in Figs. 5, 6, 7. Comparing Figs. 1 and 2 to 5, 3 to 6 and 4 to 7, it is clear that the most prescribed antibiotics do not constitute the most representative antibiotics. Specifically, the mean prediction errors with all six hospitals in Fig. 5 (solid line) and with only HUS (double line) when the three most prescribed antibiotics are used are 41.7 and 33.0, respectively, whereas the mean prediction errors with m = 3 are approximately 12–15 in Table 2.

## Discussion

Collecting data on all antibiotics is a tedious and painstaking task in Korea, and this may be the case in other countries as well. We showed that it is possible to use only 1–3 representative antibiotics to track the total antibiotic consumption at the hospital level. Our main finding is that FQ and AG are the most representative, followed by BL-BLI, 4th CEP and 3rd CEP. Our mean prediction error is only 1–3% of the monthly antibiotic consumption amount, which is the average across the hospitals and years in our data. Whether or not these levels of prediction error are tolerable depends on how much we save in terms of time and money by not collecting data on the other antibiotics.

Although our representative antibiotics were selected, not based on their medical effectiveness, but based on how well they collectively represented the total antibiotics usage, the representative antibiotics also happened to be the most commonly prescribed for inpatients, except for 4th CEP. To better monitor antibiotic consumption in hospitals, one of the broad-spectrum antibiotics or antibiotics against multi-drug resistant pathogens (such as carbapenems) could be co-monitored with our representative antibiotics.

The overall antibiotic usage patterns in our data differ little from other studies in Korea. A single-centre study found that 3rd CEP was the most commonly prescribed antibiotic for hospitalized patients in Korea, followed by FQ, BL-BLI and 1st CEP [8]. Additionally, a population-based study showed that 3rd CEP was the most prescribed antibiotic for inpatients in Korea, followed by AG, 1st CEP and FQ [9]. These studies suggest that we might have found almost the same representative antibiotics had we analysed other Korean hospitals’ data that are not in our data set.

The antibiotic usage pattern is different at various levels. For instance, in Italy and the UK, AG are not used as frequently as in Korea [10, 11], which illustrates country-level differences; also, there are large differences in the consumption profiles for treatments of the same bacterial infection among European countries [12]. Even among the hospitals in the same country, large differences in antibiotic usage patterns exist; e.g., medium-sized, private and university hospitals use more antibiotics [13]; additionally, antibiotic usage patterns differ between small and large community hospitals in Korea [9]. Possible reasons for these differences are variations in bacterial epidemiology at hospital level, the medical staff’s attitude towards prescribing antibiotics, antimicrobial stewardship programme effectiveness, etc. Hence, if possible, it would be ideal for each hospital to conduct a study of its own (as was done in this paper) to find its own representative antibiotics.

The methodology we presented used basic statistics for predicting future time-series variables. It should not be too difficult for hospitals to tailor the methodology to meet their needs, finding a few representative antibiotics by using, e.g., different functions of *t* and different structural breaks at different times. Once the methodology is set, the hospital would then address the problem of selecting a few representative antibiotics, which is in fact more difficult than it looks; e.g., if three are to be chosen out of 20 antibiotics in total, there are 1140 possible combinations. In this case, despite many differences across countries and hospitals within the same country, our findings should be helpful in choosing antibiotics to consider first (it would be FQ, AG, BL-BLI, 4th CEP and 3rd CEP); of course, the most commonly prescribed antibiotics in the hospital would also make good candidates.

We attribute the structural break in Fig. 4 at HUS to the pre-authorization of an antibiotic use programme that started in 2008. The programme put restrictions on prescribing broad-spectrum antibiotics such as carbapenems, glycopeptides, oxazolidinone, polymyxin and tigecycline by requiring an extra approval step from the infectious disease department [14]. Additionally, the programme reinforced educating physicians on the appropriate use of antibiotics and collecting feedback after drug use.

As the HUS time-series data plot illustrates in Fig. 4, a structural break can move the intercept substantially, the ignorance of which would result in large biases in the other estimates because the other estimates would be adjusted downward to account for the large drop in the intercept. Detecting structural breaks is relatively straightforward and can be accomplished by plotting the time-series data, as in Figs. 3 and 4. Of course, if the break magnitudes are small, then they are hard to detect with the naked eye, but then they would not be called “breaks”. Structural breaks might have to be incorporated using outside information such as announced law/regulation changes.

There are some notable limitations in our study. First, the six university hospitals were selected, not by any sampling principle, but by ease in data collection, in which sense our data may not be representative of the large university hospitals in Korea that would be our study population of interest. For five hospitals, we could gather only three years of data, which resulted in relatively larger standard errors than we would have liked. Second, the prediction performance was gauged using only one hospital’s single-year data, and thus, using other hospital data or a longer time span of data may alter/qualify our findings. Third, we adopted a relatively simple ordinary least squares estimator to find the time trend and monthly variations; more statistically sophisticated models and approaches may refine and improve the prediction capability. Finally, we measured antibiotic consumption by DDD instead of days of therapy (DOT). According to a recent guideline for antibiotic stewardship programmes, DOT is preferred to DDD as a measure of antibiotic consumption [15]. However, we could not use DOT because only the total amount of antibiotic consumption per patient was available in five of the six hospitals.

As far as we are aware, our study is the first of its kind to look at the possibility of using only a few antibiotics to track the total antibiotic consumption at the hospital level. Hopefully, more studies will be done to save medical personnels’ time and effort surrounding non-essential data collection, so that they can concentrate on more important healthcare activities.

## Conclusions

This study showed that the total antibiotic consumption at the hospital level can be represented sufficiently well by a few antibiotics. FQ and AG were the most representative in the sense of minimizing the mean prediction error, followed by BL-BLI, 4th CEP and 3rd CEP; the mean prediction error is only 1–3% of the monthly antibiotic consumption amount. Despite this positive finding, because our analysis is based solely on Korean data and because the medical environment/practice of each country and each hospital differs, other hospitals may follow a similar modelling strategy to find their own representative antibiotics instead of readily adopting the aforementioned antibiotics as the most representative.

## Additional file

Additional file 1: **Supplementary materials.** The model for ordinary least squares (OLS) estimator. (DOCX 97 kb)

## Abbreviations

1st CEP: 1st-generation cephalosporins
2nd CEP: 2nd-generation cephalosporins
3rd CEP: 3rd-generation cephalosporins
4th CEP: 4th-generation cephalosporins
AG: Aminoglycosides
ATC: Anatomic Therapeutic Chemical
BL-BLI: Beta-lactam/beta-lactamase inhibitors
DDD: Defined Daily Dose
DOT: Days of therapy
FQ: Fluoroquinolones
HUS: Hanyang University Seoul Hospital
OLS: Ordinary least squares
SD: Standard deviation
SE: Standard errors
WHO: World Health Organization
