# Supplementary material for: A few antibiotics can represent the total hospital antibiotic consumption
Source: BMC Infect Dis. 2018 May 31;18:247. doi: 10.1186/s12879-018-3132-7 (PMC5984315; doi:10.1186/s12879-018-3132-7)
Supplement: Supplementary file 1 — Supplementary materials. The model for ordinary least squares (OLS) estimator. (DOCX 97 kb) [file 12879_2018_3132_MOESM1_ESM.docx]

**Supplementary materials**

Our OLS model for HUS is

$$\begin{matrix} Y_{t}=\sum_{a=1}^{m} \beta_{a}X_{at} +\eta\cdot1[52\leq t] +\sum_{q=0}^{p} \alpha_{q}t^{q} \\ +\gamma_{Feb}1[t\in\text{February}]+\gamma_{March}1[t\in\text{March}]+,...,+\gamma_{Dec}1[t\in\text{December}] +error_{t} \end{matrix}$$

where the right-hand side consists of the part explained by the main antibiotics ($\sum_{a=1}^{m} \beta_{a}X_{at}$), the structural break ($\eta\cdot1[52\leq t]$), the trend ($\sum_{q=0}^{p} \alpha_{q}t^{q}$), the monthly variations relative to January, and an error. When all hospitals were used together, we dropped $\eta\cdot1[52\leq t]$, because there is no obvious break in $Y_{t}$ that is the hospital-averaged antibiotic amount.

The explanatory variables for the OLS are

$$X_{1t},..., X_{mt}, 1[52\leq t], 1, t,..., t^{p}, 1[t\in\text{February}],..., 1[t\in\text{December}].$$

The parameters estimated with these regressors are $\beta$ 's, $\eta$, $\alpha$ 's and $\gamma$ 's. As was noted in the main text, $1[52\leq t]$was used only when the HUS data were used alone.

Once the OLS was obtained, the predictor $\hat{Y}_{\tau}$ for $Y_{\tau}$ was obtained by replacing $t$ in the above equation with $\tau$ and then dropping the error term: with `^' denoting the OLS estimates,

For instance, for $\tau=109$(2013 January), we have$1[52\leq109]=1$, $1[\tau\in$ February$]=0, ...,$and $1[\tau\in$ December$]=0$, and thus

For $\tau=110$ (2013 February), we have$1[52\leq109]=1$, $1[\tau\in$ February $]=1, ...,$ and $1[\tau\in$ December$]=0$, and thus

The estimation outlined above was done with the HUS data over 2004-2012, and with all six hospital data for 2004, 2008 and 2012. In contrast, the prediction could be done only for HUS, because the 2013 data are available only for HUS. Keep in mind that the dummy variable $1[52\leq t]$ was used only when the HUS data were used alone.

One question that might arise is why the$\beta$'s were estimated, when the coefficients of $X_{at}$ 's in $\sum_{a=1}^{m} X_{at}$ were known to be one in (1). When$\beta$'s were estimated, the estimates were different from one due to the well-known ‘omitted variable bias (OVB)’ of OLS: the estimates for$\beta$'s are biased to the extent that the main antibiotics are correlated with the other antibiotics. The answer to the question is that, as long as the goal is prediction, the OVB does not matter. To the contrary, the OVB in fact helped prediction, because it occurred due to the main antibiotics accounting for the other antibiotics through their correlations.

Another question might be why the month dummies estimate the effect relative to the baseline January. To see why, let $\delta_{1}(t),..., \delta_{12}(t)$ denote the month dummies, and consider

$Y_{t}=\sum_{a=1}^{m} \beta_{a}X_{at} +\eta1[52\leq t] +\sum_{q=0}^{p} \alpha_{q}t^{q} +\sum_{j=1}^{12} \gamma_{j}\delta_{j}(t)+error.$ (3)

Since $\sum_{j=1}^{12} \delta_{j}(t)=1$ --- $t$ should equal some month---we have $\delta_{1}(t)=1-\sum_{j=2}^{12} \delta_{j}(t)$. Substituting this into (3) gives (we show only the trend and monthly variation parts)

$$\begin{matrix} \alpha_{0}+\alpha_{1}t+,...,+\alpha_{p}t^{p} + \gamma_{1}\{1-\sum_{j=2}^{12} \delta_{j}(t)\}+\sum_{j=2}^{12} \gamma_{j}\delta_{j}(t) \\ = \alpha_{0}+\alpha_{1}t+,...,+\alpha_{p}t^{p} + \gamma_{1}-\gamma_{1}\sum_{j=2}^{12} \delta_{j}(t) +\sum_{j=2}^{12} \gamma_{j}\delta_{j}(t) \\ = (\alpha_{0}+\gamma_{1})+\alpha_{1}t+,...,+\alpha_{p}t^{p} + \sum_{j=2}^{12} (\gamma_{j}-\gamma_{1})\delta_{j}(t). \end{matrix}$$

This shows that we can find the monthly variation relative to January ($\gamma_{j}-\gamma_{1}$). That is, (3) becomes

$$Y_{t}=\sum_{a=1}^{m} \beta_{a}X_{at} +\eta1[52\leq t] +\sum_{q=0}^{p} \alpha_{q}t^{q} +\sum_{j=2}^{12} \gamma_{j}\delta_{j}(t)+error$$

where $\alpha_{0}$ is redefined as $\alpha_{0}+\gamma_{1}$, and $\gamma_{j}$ as $\gamma_{j}-\gamma_{1}.$

To give a concrete idea on the OLS, we illustrate the OLS for a simple case: $m=1$ (single main antibiotic), a structural break at $t=4$, $p=2$(quadratic trend), and 17 monthly ($t=1,..., 17$) observations starting from January 2004. Let $Y\equiv(Y_{1},..., Y_{17})'$ be the $17\times1$ vector of the antibiotic amount over the 17 months that is averaged across the six hospitals. The regressor matrix $Z$ of dimension $17\times16$for the OLS is

where the first column is the $X_{1}$ amount over the 17 months, the second column is the dummy for $t=4$ or greater, and the next three columns are for $(1, t, t^{2})$. In the month dummy columns, the first dummy $\delta_{2}$ indicates February, and there are two February months in the data ($t=2, 14$). As all columns but $X_{1}$ are determined by $t$, they are free, not requiring any data collection. Using matrix notation, the OLS is $(Z'Z)^{-1}ZY$.

This OLS formula can be found in many statistics books; e.g., Draper, N.R. and H. Smith, 1998, Applied Regression Analysis, 3^rd^ ed, Wiley, and Freedman, D.A., 2009, Statistical Models: Theory and Practice, Cambridge University Press. About using t and its functions to estimate trend and seasonality, there are many time-series analysis book available; e.g., Hamilton, J., 1994, Time Series Analysis, Princeton University Press.

Table 3. Ordinary least squares estimates (OLS) for six hospitals (2004, 2008, 2012: 36 months)

|  | AG | AG + 4^th^ CEP | AG + BL-BLI + 4^th^ CEP |
| --- | --- | --- | --- |
|  | *R*^2^ = 0.918 | *R*^2^ = 0.925 | *R*^2^ = 0.928 |
| Regressor | OLS (Standard error) | OLS (Standard error) | OLS (Standard error) |
| 1 | *426.50 (211.04) | **471.95 (210.90) | *407.66 (226.17) |
| AG | **3.68 (1.42) | **3.28 (1.43) | **3.18 (1.45) |
| BL-BLI |  |  | 0.96 (1.14) |
| 4^th^ CEP |  | 2.63 (2.08) | 2.13 (2.19) |
| *t* | 2.33 (3.95) | 0.72 (4.09) | -0.19 (4.27) |
| *t*^2^/10 | 1.62 (1.07) | 1.61 (1.06) | 1.84 (1.10) |
| *t*^3^/1,000 | *-2.62 (1.45) | -2.38 (1.44) | *-2.64 (1.48) |
| *t*^4^/100,000 | 1.08 (0.65) | 0.93 (0.65) | 1.03 (0.67) |
| February | 7.65 (17.78) | 6.60 (17.52) | 6.38 (17.68) |
| March | **-38.02 (17.71) | *-35.40 (17.56) | -31.23 (18.40) |
| April | *-34.84 (18.53) | -28.56 (18.92) | -23.98 (19.85) |
| May | **-43.50 (17.94) | -30.54 (20.43) | -24.14 (21.98) |
| June | *-35.93 (18.09) | -25.52 (19.63) | -16.24 (22.69) |
| July | **-50.34 (18.43) | -35.64 (21.56) | -32.48 (22.07) |
| August | -31.68 (18.91) | -23.86 (19.62) | -18.52 (20.80) |
| September | -29.38 (18.59) | -21.66 (19.30) | -12.12 (22.56) |
| October | *-37.98 (19.92) | *-34.64 (19.79) | -24.28 (23.50) |
| November | -30.99 (20.90) | -29.64 (20.61) | -24.93 (21.53) |
| December | -29.75 (19.51) | -20.59 (20.54) | -20.62 (20.71) |

Abbreviations: 4^th^ CEP, 4th-generation cephalosporins; AG, aminoglycosides; BL-BLI, beta-lactam/beta-lactamase inhibitors. * for *P*-value<0.1; ** for *P*-value<0.05; *** for *P*-value<0.01.

Table 4. Ordinary least squares estimates (OLS) for Hanyang University Seoul hospital (2004-2012 : 108 months)

|  | FQ | AG + FQ | BL-BLI + FQ + 3^rd^ CEP |
| --- | --- | --- | --- |
|  | *R*^2^ = 0.907 | *R*^2^ = 0.917 | *R*^2^ = 0.922 |
| Regressor | OLS (Standard errors) | OLS (Standard errors) | OLS (Standard errors) |
| 1 | ***867.64 (36.73) | ***700.28 (58.76) | ***680.56 (54.07) |
| AG |  | ***1.06 (0.30) |  |
| BL-BLI |  |  | **0.62 (0.31) |
| FQ | ***1.06 (0.24) | ***1.00 (0.23) | ***1.05 (0.23) |
| 3^rd^ CEP |  |  | ***0.88 (0.21) |
| 1[52≤*t*] | ***-142.84 (17.43) | ***-97.90 (20.84) | ***-152.27 (16.53) |
| *t* | **5.28 (2.07) | ***5.30 (1.96) | 2.03 (2.05) |
| *t*^2^/10 | **-1.53 (0.72) | *-1.28 (0.68) | -0.58 (0.70) |
| *t*^3^/1,000 | *1.50 (0.85) | *1.37 (0.81) | 0.60 (0.82) |
| *t*^4^/100,000 | -0.49 (0.34) | -0.51 (0.32) | -0.22 (0.32) |
| February | 4.51 (14.76) | 9.25 (14.06) | 0.64 (13.67) |
| March | ***-52.62 (14.99) | ***-54.25 (14.22) | ***-38.33 (14.27) |
| April | *-27.60 (14.85) | **-32.59 (14.14) | -17.58 (13.98) |
| May | **-29.47 (14.85) | **-35.71 (14.19) | -10.10 (14.45) |
| June | *-26.35 (14.84) | **-29.79 (14.11) | -3.69 (14.69) |
| July | ***-43.14 (15.03) | ***-44.61 (14.26) | **-28.47 (14.27) |
| August | -18.35 (15.02) | -17.00 (14.24) | -14.52 (13.89) |
| September | **-30.29 (14.91) | **-29.53 (14.14) | -14.74 (14.47) |
| October | ***-47.78 (14.93) | ***-42.88 (14.22) | *-27.95 (14.60) |
| November | **-33.31 (15.05) | **-29.24 (14.31) | -15.22 (14.55) |
| December | -16.73 (15.04) | -11.61 (14.33) | -9.09 (13.99) |

Abbreviations: 3^rd^ CEP, 3rd-generation cephalosporins; AG, aminoglycosides; BL-BLI, beta-lactam/beta-lactamase inhibitors; FQ, fluoroquinolones. * for *P*-value<0.1; ** for *P*-value<0.05; *** for *P*-value<0.01.
